# From pathway to population – a multiscale model of juxtacrine EGFR-MAPK signalling

**DOI:** 10.1186/1752-0509-2-102

**Published:** 2008-11-26

**Authors:** DC Walker, NT Georgopoulos, J Southgate

**Affiliations:** 1 Department of Computer Science, University of Sheffield, Kroto Research Institute, North Campus, Broad Lane, Sheffield, S3 7HQ, UK; 2 Jack Birch Unit of Molecular Carcinogenesis, Department of Biology, University of York, York YO10 5YW, UK

## Abstract

**Background:**

Most mathematical models of biochemical pathways consider either signalling events that take place within a single cell in isolation, or an 'average' cell which is considered to be representative of a cell population. Likewise, experimental measurements are often averaged over populations consisting of hundreds of thousands of cells. This approach ignores the fact that even within a genetically-homogeneous population, local conditions may influence cell signalling and result in phenotypic heterogeneity.

We have developed a multi-scale computational model that accounts for emergent heterogeneity arising from the influences of intercellular signalling on individual cells within a population. Our approach was to develop an ODE model of juxtacrine EGFR-ligand activation of the MAPK intracellular pathway and to couple this to an agent-based representation of individual cells in an expanding epithelial cell culture population. This multi-scale, multi-paradigm approach has enabled us to simulate Extracellular signal-regulated kinase (Erk) activation in a population of cells and to examine the consequences of interpretation at a single cell or population-based level using virtual assays.

**Results:**

A model consisting of a single pair of interacting agents predicted very different Erk activation (phosphorylation) profiles, depending on the formation rate and stability of intercellular contacts, with the slow formation of stable contacts resulting in low but sustained activation of Erk, and transient contacts resulting in a transient Erk signal. Extension of this model to a population consisting of hundreds to thousands of interacting virtual cells revealed that the activated Erk profile measured across the entire cell population was very different and may appear to contradict individual cell findings, reflecting heterogeneity in population density across the culture. This prediction was supported by immunolabelling of an epithelial cell population grown in vitro, which confirmed heterogeneity of Erk activation.

**Conclusion:**

These results illustrate that mean experimental data obtained from analysing entire cell populations is an oversimplification, and should not be extrapolated to deduce the signal:response paradigm of individual cells. This multi-scale, multi-paradigm approach to biological simulation provides an important conceptual tool in addressing how information may be integrated over multiple scales to predict the behaviour of a biological system.

## Background

Experimentally, cell populations are usually considered to be homogeneous. Techniques such as Western Blotting inherently make the assumption that variations between individual cells are not significant. Furthermore, most computational models of signalling pathways relate to events in a 'typical' cell. Whilst adequate for most purposes, these models do not address the issue of heterogeneity – particularly in the context of how variations in the micro-environment influence gene and protein expression of individual cells.

This issue of local heterogeneity in microenvironment, and the potential influence on cell signalling and fate has yet to be studied extensively. However, recent publications have suggested that such factors may play a critical role in determining cell fate, with implications for clinically important phenomena such as stem cell lineage fate in MLL-AF9 leukaemia [1]. The architecture of intercellular contacts within beta cell islets has been shown to result in heterogenous production of insulin [2]. In this paper, we describe how we have used multi-scale computational simulations, supported by experimental data, to explore how local differences in intercellular contact may influence intracellular signalling, with implications for individual cell response and population heterogeneity.

Cell behaviour can be modelled by representing individual cells as computational entities or software agents. Unlike conventional modelling of intracellular signalling pathways, agent modelling allows the response of signalling to be interpreted at the cellular level. For example, we can incorporate the hypothetical rule that a particular threshold amount or concentration of a product is required for an individual cell to progress through the cell cycle. This method allows the study of emergent behaviour of a system (e.g. tissue growth or wound healing) as the outcome of the interaction of the individual components (the cells). We have previously developed *Epitheliome*, an agent-based model of epithelial cell populations. Software agents represent individual cells and iteratively change their state (e.g. cell cycle stage, location, shape) according to a number of pre-programmed rules representing biological behaviour, such as proliferation, intercellular adhesion, migration and apoptosis. We have used this simple rule-based model to simulate monolayer growth [3] and wound healing [4] in normal human urothelial cell cultures and have adapted it to explore stratification and differentiation in normal and transformed keratinocytes [5]. More recently, we have combined this model with a mathematical representation of autocrine ligand release, diffusion and binding [6]. The biological basis for this work has been experimentally-generated observations in cultures of normal human urothelial (NHU) cells where, in the absence of exogenous growth factors, proliferation is driven through autocrine production of EGFR-binding factors [7]. In the NHU cell culture system, inhibition either of EGFR, or of the downstream MAPK/Erk pathway components, results in dephosphorylation of Erk and an inhibition of proliferation that is reversible upon removal of the inhibitor [8]. Thus, although it is clear that multiple, interacting signalling pathways influence cell cycle progression and proliferation ([8-10]), there is compelling evidence for a causal link between EGFR-Erk signalling pathway and proliferation, at least in these cells. Autocrine growth mechanisms may operate through the release of soluble ligands, or through juxtacrine signalling mediated via cell:cell interactions and we have used a modelling approach to examine the implications of these modes of communication. Our approach of using an individual-based paradigm naturally lends itself to examining heterogeneity, either by varying the internal parameters (memory state) of each agent, or by observing the emergent behaviour of agents in differing micro-environments.

The initial realisation of the *Epitheliome *model can be considered as a phenomenological representation of actual cell behaviour, as the rules governing state transitions are based purely on observations made experimentally, many of which were extracted from the scientific literature. For instance, agents progress around the cell cycle consisting of phases representing G1, S, G2 and M, traversing a single checkpoint in G1. As described in [3], transition through this checkpoint is dependent on cell:cell bonding and cell spreading, with the underpinning rule set mimicking the phenomenon of contact inhibition of growth. Results produced by this model successfully predicted the reduction of growth rate observed experimentally when epithelial cell cultures were switched to culture conditions that promoted formation of intercellular adherens junctions, through the calcium-dependent homotypic binding of E-cadherin [3]. However, a consistent enhancement of the in vitro growth rate in low density populations grown in physiological calcium conditions was not predicted computationally, and this divergence of *in vitro *and *in virtuo *systems led us to investigate potential mechanisms for enhanced growth mediated via intercellular contact, and in particular, juxtacrine signalling via the Epidermal Growth Factor Receptor (EGFR).

EGFR is known to be a critical mediator of growth control in many cell types, including urothelium [7] and there is growing experimental evidence that EGFR ligands are biologically active prior to cleavage from the membrane [11]. In order to investigate a possible role for contact-mediated juxtacrine signalling in epithelial cell cultures, we have utilised a mathematical ordinary differential equation (ODE) model of membrane-bound EGFR-ligand interaction, receptor dimerisation/activation and downstream signalling *via *the Ras-Raf-MAPK pathway. The end point of this model pathway is the activation (diphosphorylation) of the cytoplasmic protein, Erk. It is known that activated Erk (Erk-PP) translocates to the nucleus where it can activate transcription of key cell cycle regulators, such as cyclin D1. The temporal characteristics of Erk activation have previously been shown to be closely related to cell fate, with sustained, moderate Erk activation correlated with cell cycle progression [12,13].

**Figure 1:**
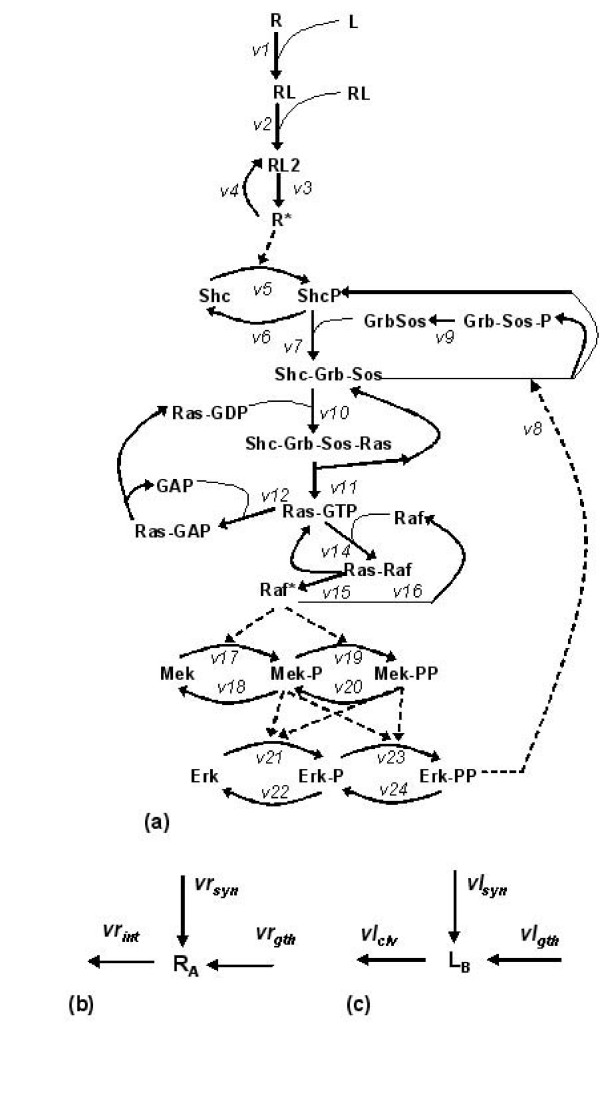
**EGFR-MAPK pathway model**. Intracellular signalling pathway model derived from [15] and [14] and b) receptor trafficking and c) ligand trafficking on the cell surface.

**Figure 2:**
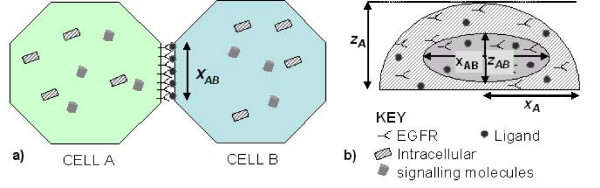
**Modelling of intercellular contact**. a) Top view and b) side view of pair of cell agents sharing an elliptical intercellular contact of length x_ab _and height z_ab_.

The Erk signalling pathway is reasonably well characterised and has been the focus of several modelling studies (e.g. [14-17]). It was not our objective to develop a new model of this pathway, but to incorporate an existing mathematical description into our agent-based representation of cells, hence introducing a subcellular level 'mechanism' that is initiated by cellular scale interactions to influence emergent changes in cell behaviour. We have thus chosen to integrate and adapt existing models of this pathway [14,15] in order to consider activation of the Erk pathway in an individual cell which occurs as a result of regions of intercellular contact with one or more neighbouring cells, through activation of EGFR by cell surface-presented cognate ligands. These intercellular regions can be fixed in size, or actively growing in accordance with the reported behaviour of E-cadherin mediated contacts in cultured epithelial cells [18]. The various components of the model, and our methodology in integrating them are described in the Methods section and illustrated in Figure 1, 2, 3. We present the simulated time-varying activated Erk (Erk-PP) profile for the following scenarios: 1) a pair of cells forming a single, transient or stable contact (the latter being typical of E-cadherin-mediated adherens junctions) and 2) the ERK-PP profile averaged over a growing population of several hundred to thousands of agents in low and physiological calcium concentrations, (the latter conditions being conducive for the formation of multiple, stable E-cadherin-mediated contacts). The multi-agent simulations inherently include inter-agent microenvironment heterogeneity, as this reflects variation in the nature of intercellular contacts experienced by individual cell agents as a result of their position within the population. We also present the results of a sensitivity analysis of parameters in the signalling pathway model.

**Figure 3:**
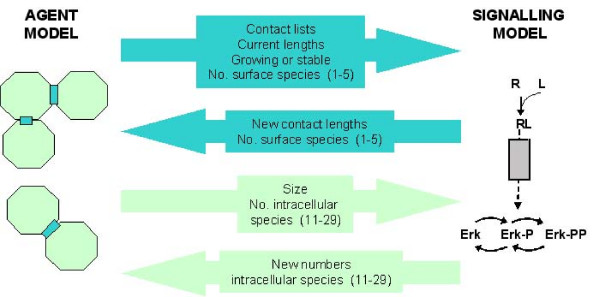
**Model flow**. Information passed between agent and signalling model. Blue arrows indicate information passed per contact, green arrows represent information passed per agent.

## Methods

### EGFR-Erk signalling model

The primary criteria for selecting a suitable model of intracellular signalling for integration into our existing modelling framework were: 1) ability of the model to simulate the key features of the output (temporal changes in Erk-PP expression) with respect to input (receptor activation), 2) minimum complexity (to introduce parsimony by restricting the number of ODEs required for future multi-agent and hence multi-contact simulations), and 3) inclusion of key stages from the entire pathway, as opposed to a limited subset, to allow future mapping of signal to cellular response.

On the basis of these criteria, the model chosen as a starting point was that of [14], which was originally used to examine the temporal pattern of Erk activation in response to soluble EGF or NGF binding to surface receptors. This model was selected due to its relative simplicity compared to other more detailed descriptions, although this does not impact on its ability to predict key features of the pathway, notably the high amplitude transient peak in activated Erk, followed by a lower, but sustained activation for periods of several hours. The receptor-ligand dynamics were slightly simplified and the equations used for this part of the model were those implemented in [15]. A schematic of the kinetic model is shown in figure 1a.

For the purposes of this study, we consider the case where the presence of intercellular contacts means that receptor activation can occur only on a limited region of the cell surface and thus involve only a restricted number of receptors and ligands. We ignore the role of soluble ligands, assuming that the cells are in a growth medium that does not incorporate exogenous EGF ligands. Receptors located in the region of any intercellular contact can be subject to internalisation, but with surface expression maintained by receptor recycling or *de novo *receptor synthesis. In addition, the growth of contact areas between cells, such as that observed during E-cadherin mediated adhesion, will result in additional receptors entering the contact area. Similarly, ligands will increase in number due to synthesis, or increases in contact area, whereas they are lost by proteolytic cleavage (as shown in figure 1b–c). Taking all these processes into account, the equations describing receptors in cell A and ligands on cell B associated with a shared intercellular contact of length *x*_*AB *_and height *z*_*AB *_are given by equations (1) and (2) in table 1. Cells are considered to be oblate hemi-spheroids (as shown in figure 2). Contact areas are considered to be elliptical in shape, with the maximum height of a contact, *z*_*AB*_, fixed at 5 μm (a reasonable value considering the flattened morphology of adherent epithelial cells). The length of E-cadherin mediated contacts grow linearly with time until a maximum length of 1.5 times the cell radius is reached (as specified by equation 6 in table 2). This maximum threshold is based on the examination of phase contrast microscopy images of growing urothelial cell cultures. E-cadherin contacts will not expand further if >95% of the cell perimeter is already associated with intercellular contacts.

**Table 1:** EGFR signalling and trafficking.

	**Species**	**Rate Equation**	**Constants**	**Ref**
1	R_A_	$\frac{{\text{d[R}}_{\text{A}}]}{\text{dt}}=-{\text{ k}}_{\text{1}}{\text{[R}}_{\text{A}}{\text{][L}}_{\text{B}}\text{]}+{\text{k}}_{-\text{1}}{\text{[RL}}_{\text{A}}\text{]}-{\text{k}}_{\text{e}}{\text{[R}}_{\text{A}}\text{]}+\frac{{\text{k}}_{\text{Rsyn}}.\pi .x_{AB}.z_{AB}}{4SA_{A}}+\Omega_{AB}\frac{.\pi .z_{AB}\sigma .{\left[R0\right]}_{A}}{4SA0_{A}}$	k_1 _= 3 × 10^-4^ k_-1 _= 0.23 k_e _= 0.03 k_Rsyn _= 300	[25] [31]
2	L_B_	$\frac{{\text{d[L}}_{\text{B}}]}{\text{dt}}=-{\text{k}}_{\text{1}}{\text{[R}}_{\text{A}}{\text{][L}}_{\text{B}}\text{]}+{\text{k}}_{-\text{1}}{\text{[RL}}_{\text{A}}\text{]}-{\text{k}}_{\text{clv}}{\text{[L}}_{\text{B}}\text{]}+\frac{{\text{k}}_{\text{Lsyn}}.\pi .x_{AB}.z_{AB}}{4SA_{B}}+\Omega_{AB}\frac{.\pi .z_{AB}\sigma .{\left[L0\right]}_{B}}{4SA0_{B}}$	k_Lclv _= 0.005 k_Lsyn _= 250	[31-33]
3	RL_A_	$\frac{{\text{d[RL}}_{\text{A}}]}{\text{dt}}={\text{k}}_{\text{1}}{\text{[R}}_{\text{A}}{\text{][L}}_{\text{B}}\text{]}-{\text{k}}_{-\text{1}}{\text{[RL}}_{\text{A}}\text{]}-{\text{2k}}_{\text{2}}[RL_{A}][RL_{A}]+2k_{-2}[RL2_{A}]$	k2 = 0.001 k_2 = 6.0	[15]
4	RL2_A_	$\frac{{\text{d[RL2}}_{\text{A}}]}{\text{dt}}={\text{2k}}_{\text{2}}[RL_{A}][RL_{A}]-2k_{-2}[RL2_{A}]-k_{3}[RL2_{A}]+k_{-3}[RP_{A}]$	k3 = 60 k_3 = 0.6	[15]
5	RP_A_	$\frac{{\text{d[RP}}_{\text{A}}]}{\text{dt}}=k_{3}[RL2_{A}]-k_{-3}[RP_{A}]-\frac{V_{4}[RP_{A}]}{K_{4}+[RP_{A}]}-k_{\mathrm{int}}[RP_{A}]$	V4 = 2.3 × 10^6^ K4 = 3 × 10^4^ kint = 0.19	[25]

Equations relating to juxtacrine receptor – ligand interaction and receptor dimerisation and phosphorylation. Constants are either extracted directly from, inferred from or reported in the cited references. Units – All parameters relate to numbers of molecules – square brackets are for clarity only. Units are: first order rate constant: min^-1^; second order rate constants molecules^-1 ^min^-1^, maximal enzyme rates (V_max_) are expressed in units of molecules min^-1^, Michaelis constants (K_m_) in molecules. k_Rsyn _and k_Lsyn _are in no. molecules cell^-1 ^min^-1^. Conversions from concentrations to numbers of molecules assume a cell volume of 10^-12 ^litres.

**Table 2:** Intercellular contact dynamics.

	**Species**	**Rate equation**	**Constants**	**Refs**
6	x_ab_	$\frac{dx_{ab}}{dt}=\sigma$	σ = 3.5/60 μm min^-1^	[18]
7	R0_A_	$\frac{d[R0_{A}]}{dt}=-\sum_{cnt=1}^{n}\Omega_{A}\frac{.\pi .z_{AB}\sigma .{\left[R0\right]}_{A}}{4SA0_{A}}-{\text{k}}_{\text{e}}{\text{[R0}}_{\text{A}}\text{]}+\frac{{\text{k}}_{\text{Rsyn}}[SA0_{A}]}{4SA_{A}}$		
8	SA0_A_	d[SA0]Adt−∑cnt=1nΩA.π.zABσ4		
9	LO_B_	$\frac{d[L0_{B}]}{dt}-\sum_{cnt=1}^{m}\Omega_{B}\frac{.\pi .z_{AB}\sigma .{\left[L0\right]}_{B}}{4SA0_{B}}{\text{ -k}}_{\text{clv}}{\text{[L0}}_{\text{B}}\text{]}+\frac{{\text{k}}_{\text{Lsyn}}[SA0_{B}]}{4SA_{B}}$		
10	SA0_B_	$\frac{d[SA0_{B}]}{dt}-\sum_{cnt=1}^{m}\Omega_{B}\frac{.\pi .z_{AB}\sigma }{4}$		

Rate equations relating to length of intercellular contact and numbers of receptors and ligands not associated with regions of contact.

The first three terms on the right hand side of the equations (1) and (2) in table 1 represent changes in the numbers of species arising from receptor-ligand interactions. The fourth term represents receptor internalisation or ligand cleavage, the fifth represents receptor/ligand synthesis and the last terms describe recruitment of new receptors or ligands into expanding contact areas. The constant *k*_1 _is the receptor-ligand association constant; *k*_-1 _the dissociation constant; *k*_*int *_the rate of internalisation of unoccupied receptors, *k*_*Rsyn *_and *k*_*Lsyn*_, the rates of *de novo *receptor and ligand synthesis respectively per cell; *SA *denotes the total surface area of the cell; σ is the rate constant for E-cadherin mediated contact growth; *R*_0_, *L*_0 _and *S*_*A*0 _are the total receptor or ligand numbers and surface area on the cell not associated with intercellular contacts at any given time (given by equations 7–10 in table 2); and Ω_*AB *_is a flag indicating active contact growth, where Ω_*AB *_= 1 if the contact is defined to be expanding in size, and = 0 otherwise. Diffusion of EGFR and ligands in and out of the contact area is ignored. This is justified by the observation that typical diffusivities are extremely small (of the order of 10^-10 ^cm^2^/second), and that movement of surface EGF receptors is restricted by the cytoskeletal network [19].

The number of occupied, dimerised and phosphorylated (active) receptors associated with contact *AB *on cell *A *will then be given by equation (5) in table 1. Similar equations will exist for EGF receptor-associated species on cell *B *associated with ligands on cell *A*.

Each cell will have *n *sets of equations 1–5, each associated with an existing intercellular contact i.e. the total activated receptor number is calculated on a per contact basis. It is assumed that a cell can form a maximum of 6 contacts. The *n *sets of equation 5 are then summed to provide the total number of activated receptors per cell (*RP *in equation 11–12, table 3), which describes the conversion of the adaptor protein Shc to ShP). All downstream species are then calculated on a per cell basis.

**Table 3:** Intracellular pathway model. Rate equations relating to intracellular EGFR-MAPK pathway.

	**Species**	**Rate equation**	**Constants**
11	Shc	$\frac{\text{d[Shc]}}{\text{dt}}=-{\text{k}}_{\text{5}}\sum_{c\text{nt}=\text{1}}^{\text{na}}\text{[RP]}{\text{[Shc]/(K}}_{\text{5}}+\text{[Shc])}+{\text{V}}_{\text{6}}{\text{[Shc-P]/(K}}_{\text{6}}+\text{[ShcP])}$	*k*_5 _= 12 *K*_5 _= 6 × 10^3^
12	ShcP	$\begin{array}{l} \frac{\text{d[ShcP]}}{\text{dt}}=\frac{{\text{k}}_{\text{5}}\sum_{c\text{nt}=\text{11}}^{\text{na}}\text{[RP]}\text{[Shc]}}{{\text{K}}_{\text{5}}+\text{[Shc]}}-\frac{{\text{V}}_{\text{6}}\text{[ShcP]}}{{\text{K}}_{\text{6}}+\text{[ShcP]}}-{\text{k}}_{\text{7}}\text{[ShcP][GrbSos] }\\ +{\text{k}}_{-\text{7}}\text{[ShcGrbSos]}+\frac{{\text{ k}}_{\text{8}}\text{[ErkPP][ShcGrbSos]}}{{\text{K}}_{\text{8}}+\text{[ShcGrbSos]}} \end{array}$	*V*_6_= 3.0 × 10^5^ *K*_6 _= 6 × 10^3^ *K*_7 _= 2 × 10^-3^ *k*_-7 _= 3.81
13	GrbSos	d[GrbSos]dt=−k[ShcP]7[GrbSos]+k−7[ShcGrbSos]+V9[GrbSosP](K9+[GrbSosP]	*K*_8 _= 1.6; *K*_8 _= 6 × 10^5^
14	GrbSosP	$\frac{\text{d[GrbSosP]}}{\text{dt}}=\frac{{\text{k}}_{\text{8}}\text{[ErkPP][ShcGrbSos] }}{{\text{K}}_{\text{8}}+\text{[ShcGrbSos]}}-\frac{{\text{V}}_{\text{9}}\text{[GrbSosP]}}{{\text{K}}_{\text{9}}+\text{[GrbSosP]}}$	*V*_9 _= 75; *K*_9 _= 2.0 × 10^4^
15	ShcGrbSos	d[ShcGrbSos]dt=k[ShcP]7[GrbSos]−k−7[ShcGrbSos]−k8[ErkPP][ShcGrbSos] K8+[ShcGrbSos]−k10[RasGDP][ShcGrbSos]+k−10[ShcGrbSosRas]+k11[ShcGrbSosRas]	*k*_10 _= 1.63 × 10^-2^ *k*_10 _= 10 *k*_11 _= 15
16	RasGDP	$\frac{\text{d[RasGDP]}}{\text{dt}}=-k_{10}[RasGDP][ShcGrbSos]+k_{-10}[ShcGrbSosRas]+k_{13}[RasGAP]$	*k*_12 _= 5.0 × 10^-3^ *k*_-12 _= 60
17	ShcGrbSosRas	$\frac{\text{d[ShcGrbSosRas]}}{\text{dt}}=k_{10}[RasGDP][ShcGrbSos]-k_{-10}[ShcGrbSosRas]-k_{11}[ShcGrbSosRas]$	k_13 _= 7.2 × 10^2^ *k*_14 _= 1.2 × 10^-3^
18	RasGTP	$\begin{array}{l} \frac{\text{d[RasGTP]}}{\text{dt}}={\text{k}}_{-\text{11}}\text{[ShcGrbSosRas]}-{\text{k}}_{\text{12}}\text{ [RasGTP] [GAP]}+\\ {\text{k}}_{-\text{12}}\text{[RasGAP]}-{\text{k}}_{14}\text{[Raf][RasGTP]}+{\text{k}}_{-\text{14}}\text{[RasRaf ]}+{\text{k}}_{\text{15}}\text{[RasRaf]} \end{array}$	*k*_-14 _= 3.0 *k*_15 _= 27
19	GAP	$\frac{\text{dGAP]}}{\text{dt}}=-{\text{k}}_{\text{12}}\text{[RasGTP][GAP]}+{\text{k}}_{-\text{12}}\text{[RasGAP]}+{\text{k}}_{13}[RasGAP]$	*V*_16 _= 9.7 × 10^4^ *K*_16 _= 6 × 10^3^
20	RasGAP	$\frac{\text{d[RasGAP]}}{\text{dt}}={\text{k}}_{\text{12}}\text{[RasGTP][GAP]}-{\text{k}}_{-\text{12}}\text{[RasGAP]}-{\text{k}}_{13}[RasGAP]$	*k*_17 _= 50; *K*_17 _= 9 × 10^3^
21	Raf	$\frac{\text{d[Raf]}}{\text{dt}}=-{\text{k}}_{\text{14}}\text{[RafP][RasGTP]}+{\text{k}}_{-\text{14}}\text{[RasRaf]}+\frac{{\text{V}}_{\text{16}}[RafP]}{{\text{K}}_{\text{16}}+[RafP]}$	*V*_18 _= 9.2 × 10^5^ *K*_18 _= 6 × 10^5^
22	RasRaf	$\frac{\text{dRasRaf]}}{\text{dt}}={\text{k}}_{\text{14}}\text{[RafP][RasGTP]}-{\text{k}}_{-\text{14}}\text{[RasRaf]}-{\text{k}}_{\text{15}}[RasRaf]$	*k*_19 _= 50; *K*_19 _= 9 × 10^3^
23	RafP	$\frac{\text{d[RafP]}}{\text{dt}}=k_{15}[RasRaf]-\frac{{\text{V}}_{\text{16}}[RafP]}{{\text{K}}_{\text{16}}+[RafP]}$	*V*_20 _= 9.2 × 10^5^ *K*_20 _= 6 × 10^5^
24	Mek	$\frac{\text{d[Mek]}}{\text{dt}}=\frac{-{\text{k}}_{\text{17}}\text{[RafP][Mek] }}{{\text{K}}_{\text{17}}+[\text{Mek]}}+\frac{{\text{V}}_{\text{18}}[MekP]}{{\text{K}}_{\text{18}}+[MekP]}$	*k*_21 _= 8.3; *K*_21 _= 9 × 10^4^
25	MekP	$\frac{\text{d[MekP]}}{\text{dt}}=\frac{{\text{k}}_{\text{17}}\text{[RafP][Mek] }}{{\text{K}}_{\text{17}}+[\text{Mek]}}-\frac{{\text{V}}_{\text{18}}[MekP]}{{\text{K}}_{\text{18}}+[MekP]}\frac{-k_{19}[\text{RafP}][\text{MekP}]}{K_{19}+[\text{MekP}]}+\frac{V_{20}[MekPP]}{K_{20}+[MekPP]}$	*V*_22 _= 2.0 × 10^5^
26	MekPP	$\frac{\text{d[MekPP]}}{\text{dt}}=\frac{{\text{k}}_{\text{19}}\text{[RafP][MekP] }}{{\text{K}}_{\text{19}}+[\text{MekP]}}-\frac{{\text{V}}_{\text{20}}[MekPP]}{{\text{K}}_{\text{20}}+[MekPP]}$	*K*_22 _= 6 × 10^5^ *k*_23 _= 8.3;
27	Erk	$\frac{\text{d[Erk]}}{\text{dt}}=\frac{-{\text{k}}_{\text{21}}\left(\text{[MekP]}+\text{[MekPP]}\right)\text{[Erk] }}{{\text{K}}_{\text{21}}+[\text{E}rk\text{]}}+\frac{{\text{V}}_{\text{22}}[ErkP]}{{\text{K}}_{\text{22}}+[ErkP]}$	*K*_23 _= 9 × 10^4^
28	ErkP	$\begin{array}{l} \frac{\text{d[ErkP]}}{\text{dt}}=\frac{{\text{k}}_{\text{21}}\left(\text{[MekP]}+\text{[MekPP]}\right)\text{.[Erk] }}{{\text{K}}_{\text{21}}+[\text{E}rk\text{]}}-\frac{{\text{V}}_{\text{22}}[ErkP]}{{\text{K}}_{\text{22}}+[ErkP]}\\ -\frac{{\text{k}}_{\text{23}}\left(\text{[MekP]}+\text{[MekPP]}\right)\text{.[ErkP] }}{{\text{K}}_{\text{23}}+[\text{E}rkP\text{]}}+\frac{{\text{V}}_{\text{24}}[ErkPP]}{{\text{K}}_{\text{24}}+[ErkPP]} \end{array}$	*V*_24 _= 4.0 × 10^5^; *K*_24 _= 6 × 10^5^
29	ErkPP	$\frac{\text{d[ErkPP]}}{\text{dt}}=\frac{{\text{k}}_{\text{23}}\left(\text{[MekP]}+\text{[MekPP]}\right)\text{.[ErkP] }}{{\text{K}}_{\text{23}}+[\text{E}rkP\text{]}}-\frac{{\text{V}}_{\text{24}}[ErkPP]}{{\text{K}}_{\text{24}}+[ErkPP]}$	

Units are as for Table 1. All constants are taken directly from [14].

The intracellular pathway associated with each cell is thus represented by 19 ODEs (equations 11–29, table 3), with individual steps represented by standard mass action kinetics, or, where appropriate, Michaelis-Menten kinetics [14]. This model was solved using the Mathworks Matlab *ode23tb *solver.

### Multiscale Modelling

The agent (rule-based) and signalling (ODE-based) models are run alternately. Each agent model iteration represents a total period of 30 minutes and the total solution period of the following call to the ODE model represents the same 30 minute period. The data passed between the models on a *per contact *and *per agent *basis is summarised in figure 3. It is assumed that agent sizes and positions are static during the solution of the signalling model (though existing contacts can grow in length). The creation or destruction of contacts is determined entirely by the agent model, so the minimum length of time that a transient (non E-cadherin stabilised) contact exists is 30 minutes. This is reasonable, given that a detailed study of high-resolution time-lapse images of urothelial cells grown in low calcium conditions (0.09 mM) suggested that most transient intercellular contacts persist between 10 and 60 minutes (e.g. additional files 1 and 2). At the end of every simulation, the updated profile of Erk-PP (equation 29) is returned to the memory of each agent. Numbers of all intracellular molecular species (11–29) are stored for every agent and surface species (1–5) for every contact, in order to provide the initial conditions for the next set of calculations. The identities of cells sharing any contact are also stored. In the event that a pre-existing contact is registered as broken following the subsequent agent iteration (a frequent occurrence for non-E-cadherin-mediated cell contacts), the receptor and ligand terms in equations (1) and (2), and equivalent equations for cell B, are set to zero and the ODE solution deals with residual intracellular signalling only.

The model described above was used to study differences in the activated EGFR-mediated conversion of Erk into its active diphosphorylated form (active Erk or Erk-PP) associated with the differing nature of contacts seen in low (0.09 mM) and near physiological (2 mM) calcium environments. This is considered to be the primary 'output' of the model, due to its role in mediating cell behaviour [12,13]. Simulations were initially carried out for the simplified case of a single pair of agents sharing a transient or E-cadherin-mediated contact. This concept was then extended to a much larger model consisting of hundreds to thousands of agents forming contacts on a stochastic basis.

### Multi-agent simulations

The basis for this component of the computational model is the prototype Epitheliome model, running on the Mathworks Matlab platform, as described in detail in [3,4]. Briefly, individual cells are represented by software agents (instances of class objects in object-oriented Matlab). Each agent contains memory parameters representing its current state (e.g. location, size and cell cycle position). The simulation is run iteratively with each agent updating its memory parameters ('state') according to a number of pre-programmed rules representing biological behaviour such as proliferation, intercellular adhesion, migration and apoptosis. Parameters are scaled such that each model iteration represents 30 minutes in real time. A numerical algorithm is employed after each agent update to shift the cells to minimise edge overlap arising due to cell growth, division and migration. We refer the reader to our earlier publication for a comprehensive description of basic model operation and the details of this numerical algorithm [3].

In order to focus on modelling cellular interaction, and in particular, biochemical signalling processes that might be influenced by direct cellular contact, it was necessary to increase the complexity of rule sets governing migratory and adhesive behaviour. These changes are described below:

#### i) E-cadherin-mediated contacts

The cadherins are a family of transmembrane proteins that mediate intercellular adhesion via interactions between homotypic proteins on opposing cell surfaces. Epithelial tissues express the cadherin subgroup, E-cadherin, which is critical for forming initial adherens contacts between cells in developing tissues or cell cultures, allowing more established structures such as tight junctions and desmosomes to develop. In order to assume a functional conformation, E-cadherin requires the presence of extracellular calcium ions. It has been demonstrated that intercellular contacts form in cultures of normal human urothelial (NHU) cells at a critical calcium concentration ≥ 1 mM [20]. A sigmoidal relationship between bonding activity and exogenous calcium, with the inflection point located at 1 mM was measured in doublets of Chinese hamster ovary cells [21].

##### Adhesion probability

We have extended our previous, simple sigmoidal relationship between extracellular calcium and E-cadherin-mediated cell contact to a more sophisticated rule set which more quantitatively reflects the observations in [21,22]. Each agent has a parameter representing normal endogenous E-cadherin expression, which varies between 1 (normal expression), to zero (no expression – as for example, found in malignant epithelial cells). This is converted to an amount of functional surface E-cadherin by the relationship:

$$P=A+\frac{C}{{\left(1+Te^{-B(X-M)}\right)}^{\frac{1}{T}}}$$

where *P *= amount of functional E-cadherin, *A *= lower asymptote (= 0.1), *C *= upper asymptote (= 0.57), *M *= point of max growth (= 1 mM), *B *= growth rate (= 1), *T *determines relationship between max growth and asymptotes (= 1) and *X *is the exogenous calcium concentration in mM. The relationship between functional E-cadherin amount, *P *and exogenous calcium thus closely mirrors the relationship described by [21] between binding activity and calcium concentration.

The bonding probability *BP*, between pairs of cells at a maximum separation distance of 5 μm (estimated to correspond to maximum length of lamellipodia extension) is then calculated:

$$BP=\frac{\sqrt{\left(P_{1}*P_{2}\right)}}{{\left(esep+1\right)}^{\frac{1}{3}}}$$

The pair of cells will form an initial E-cadherin mediated contact if a pseudo-random number is less than *BP*.

##### E-cadherin-mediated bond length

Previously, we have modelled intercellular bonds simply as being either present or absent. However, in order to explore juxtacrine signalling, it was necessary to incorporate the concept of a finite bond length. On forming an initial contact, E-cadherin molecules become aggregated, leading to the 'zippering' of adjacent cell membranes over a number of hours [18]. We devised a rule dictating the increase in bond length with time from initial contact formation, based on a quantitative interpretation of these results:

   ***for (0.5 < t < 12)***

      BL = 2 + (σ *t)

   ***else***

      ***BL = 15;***

where *BL *= bond length and *t *= time in hours and σ = 3.5 μm/hour. As this linear expansion of contacts occurs on a similar timescale as the intracellular signalling dynamics, this 'rule' is incorporated into the signalling model (equation 6, table 2).

#### ii) Transient cell-cell interactions

Close inspection of time-lapse microscopy images of NHU cells cultured in low calcium medium (0.09 mM) revealed that although significantly fewer stable contacts are formed than in physiological calcium, cells tended to remain in contact with one another for periods of up to one hour, before migrating away to form new contacts (see additional files 1 and 2). During this period of transient contact, it is feasible that juxtacrine signals may be exchanged between cells and therefore, we have chosen to include the concept of transient cell contact in our agent model. These could represent weak interactions mediated by inter-membrane surface tension, as implied by immunofluorescence microscopy (see [20], and additional files 1 and 2). We have included the rule:

*for cells with edge separation, sep, less than 0.1 μm*

   ***if sep < 0***

      ***clen = max_r;***

   ***else***

      clen = (0.1-sep)*max_r/0.1;

where *clen *is the length of the non-specific contact length and *max_r *is 1.5 times the larger of the two cell radii The form of this relationship is somewhat arbitrary, but the range of contact lengths produced gives a reasonable agreement with that observed in microscopy images of growing NHU cell cultures ([20], additional file 1). Note that cell agents in low calcium are also permitted to form E-Cadherin mediated contacts, but according to the rules described above, these occur with a much lower frequency than in higher calcium environments.

#### iii) Migration rules

In our earlier agent models (e.g. [3,6]), only cells with zero intercellular contacts were able to migrate. Introduction of the new bond probability rules and the concept of transient contacts described above resulted in very limited migration even in low calcium conditions; this situation did not mirror that observed in real NHU cell cultures. Hence, new rules were introduced that determined the ability of an agent to migrate to be the outcome of the difference between the active migration force (reported to be approximately 20 nN [23]) and intercellular adhesion force resulting from stable and transient cell contacts (reported in the former case to be approximately 100 nN after 30 minutes, and 200 nN after one hour of intercellular contact for normal E-cadherin expression levels, and linearly related to the square of the amount of E-cadherin present at the cell surface [22]). In the case of transient contacts, surface tension between non-adherent cells was calculated to be approximately 0.32 nN per micrometre of boundary in contact [24]. This leads to the following formulation for total adhesion force acting on a cell:

*AF *= (0.32 * *clen*) + (*P*_1 _* *P*_2_(100 * *b*1 + 200 * *b*2))

where *clen *is the length of cell boundary involved in non specific contacts, *b1 *and *b2 *are the number of E-cadherin mediated bonds that have existed for less and more than one hour respectively, and *P1 *and *P2 *are the levels of functional E-cadherin expression (between 0 and 1).

The model rule implemented was that a total intercellular adhesion force less than 20 nN would result in migration of a particular cell, but for larger values the cell was not permitted to migrate. For normal levels of functional E-cadherin expression, this effectively means that cells with one or more E-cadherin contacts were prevented from migration (as was the case in the earlier versions of our model), whereas those with contacts defined as transient were permitted to migrate only if the total boundary length involved is less than 62.5 μm. The maximum migration velocity was estimated from time-lapse images of NHU cells to be in the region of 80 μm/hour.

## Results

### Phosphorylated-Erk profile associated with a single intercellular contact

**Figure 4:**
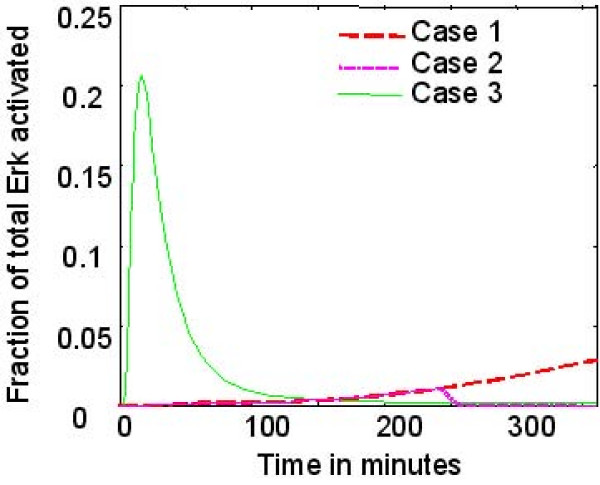
**Results of intracellular signalling model**. Model predictions – effect of cell contact stability on Erk-PP. Case 1, two cells make an initial small contact of 2 μm, which increases linearly to 23 μm over 6 hours. Case 2 shows the same initial formation pattern, but with an instantaneous break of contact after 4 hours when it has reached 16 μm length. Case 3 shows the signal arising from an 'instantaneously' formed 12 μm contact.

Figure 4 shows the predicted active Erk signal resulting from juxtacrine EGFR-ligand engagement and subsequent intracellular signalling when two cells (each assumed to be oblate hemispheres with horizontal radius = 20 μm, and vertical height = 10 μm) form contacts at different rates (as illustrated in figure 2). In case 1, the cells make an initial small contact of 2 μm, which increases linearly, as observed in [18], until a maximum length of approximately 23 μm is reached after 6 hours. Case 2 shows the same initial formation pattern, but in this case there is an instantaneous break of contact after 4 hours when it has reached 16 μm length. Finally, for comparison, case 3 shows the signal arising from an 'instantaneously' formed 12 μm contact (typical of those we have observed using time-lapse microscopy between migratory cells maintained in medium with a low (sub-physiological; 0.09 mM) calcium concentration). Results show a strong dependence of the active Erk signal on the rate of contact formation, with moderately large, instantly formed contacts (Case 3) resulting in a large peak in Erk-PP occurring approximately 20 minutes after the initial contact is made, which rapidly declines to very low levels (<0.3%), even though the contact persists. By contrast, when a contact is formed gradually (Case 1–2), this results in a slow but steady increase in Erk activation, with a contact that represents ~7% of the total surface area of the cell resulting in ~3% Erk activation. If the contact is lost, as shown in case 2, the Erk-PP signal quickly decays to zero in a matter of minutes.

A potential flaw of many computational models is hyper-sensitivity to one or more parameters. This can be particularly problematic when the parameters are difficult to measure experimentally and are thus estimated or assumed. This is a critical issue in multi-scale models such as that described here, where sensitivity to parameters at one model scale could potentially propagate through into higher scales, often with unforeseen effects.

In order to assess the sensitivity of the results described above to the model parameters, a sensitivity analysis was carried out as follows. Each of the 50 parameters in the signalling pathway model was varied systematically by first dividing, then multiplying the value of each parameter by a factor of two. The reason for selecting a relatively large perturbation factor was that the kinetic parameters for the upstream pathway model were derived from radio-labelling studies utilising the soluble form of an EGFR binding ligand (e.g. [25]) and parameters for membrane-bound receptor-ligand interactions might be expected to vary quite significantly.

The signalling pathway model was solved for both a transient, 15 μm contact and a growing then stable 3 μm contact. A sensitivity value S was calculated for each case in terms of the maximum amplitude and duration (defined as the length of time where the signal is greater than 10% of its maximum value):

$$S=\frac{\text{fractional change in output parameter}}{\text{fractional change in input parameter}}$$

**Figure 5:**
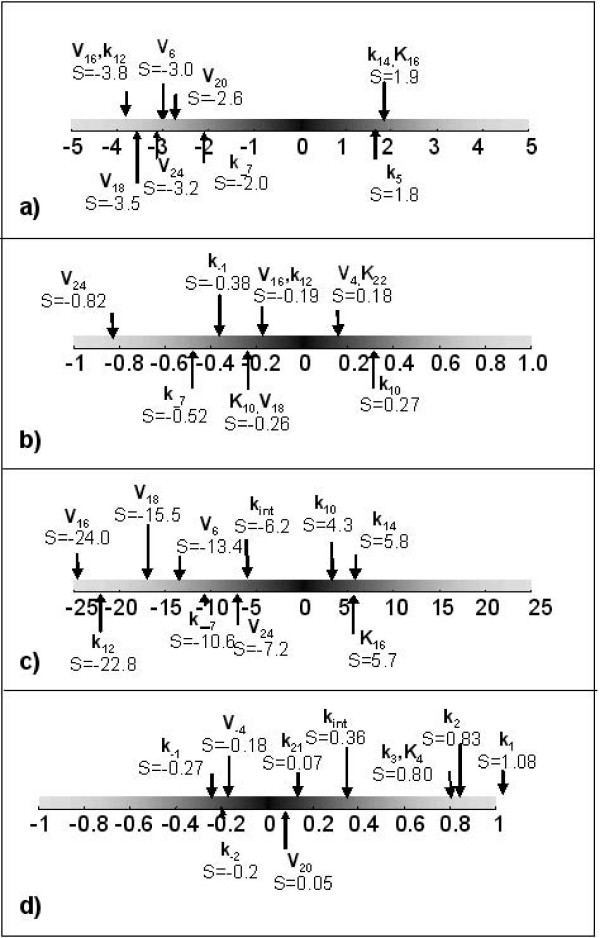
**Results of sensitivity analysis**. Diagrammatic representation of results sensitivity analysis of signalling pathway model a) maximum amplitude of Erk-PP associated with transient contact b) duration of Erk-PP associated with transient contact c) maximum amplitude of Erk-PP associated with growing then stable contact d) duration of Erk-PP associated with growing then stable contact.

The distribution of the ten largest values of S for both contact types is shown in figure 5. Although there is variation in the amplitude and duration of the ERK-PP signals when kinetic parameters are altered, none of the parameter variations affected the fundamental result that transient contacts result in a relatively large, transient response, compared to the smaller but sustained response associated with slowly-growing stable contacts. The durations of the signals were relatively insensitive to the model parameters. Interestingly, the parameters associated with the highest sensitivity values were typically those describing dephosphorylation reactions in the downstream section of the pathway. For instance, *V*_16_*, V*_18 _*and V*_24_, (which describe the maximal rate of Raf, Mek and Erk dephosphorylation respectively – see table 3), all had a significant influence on signal amplitude; by contrast, parameters describing the rate of receptor ligand binding, receptor dimerisation, activation and internalisation had a much less significant effect. These results suggest that the inclusion of rate constants that have been derived from interaction of soluble ligands with cell surface receptors in a model of juxtacrine signalling is not likely to be the largest cause of error and uncertainty in the model, and irrespective of the exact values of these constants, the temporal pattern of Erk activation is likely to depend on the nature of intercellular contacts.

### Modulation of intercellular contacts by calcium concentration

Extracellular calcium is known to be a key mediator of intercellular contacts, with concentrations greater than 1 mM resulting in stable adherens junctions mediated by the homotypic interaction of E-Cadherin molecules expressed on the membranes of adjacent cells [21]. Additionally, cells may form short-term or transient contacts that are independent of the exogenous calcium concentration (for example, see additional files 1 and 2).

**Figure 6:**
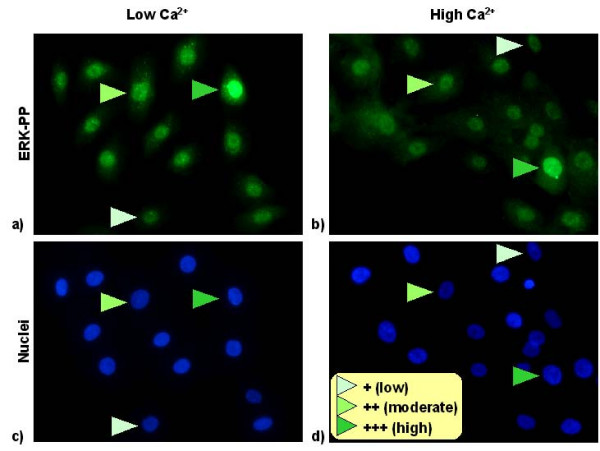
**Erk-PP immunofluoresence results**. Distribution of fluorescently tagged Erk-PP in normal human urothelial cells maintained in low 0.09 mM (a) and physiological 2.0 mM (b) calcium. c) and d) show position of nuclei.

Testing the agent-only model with the rule sets described in Methods was found to give an increased incidence of E-cadherin-mediated bonding in low calcium conditions than we have described in our previous modelling studies. Comparison of simulated E-cadherin-mediated contact incidence at different cell densities gave good qualitative agreement with the results of an E-cadherin immunofluorescence microscopy study carried out using cultured NHU cells maintained in growth media containing 0.09 mM and 2.0 mM (physiological) calcium concentrations, respectively, as shown in figure 6.

**Figure 7:**
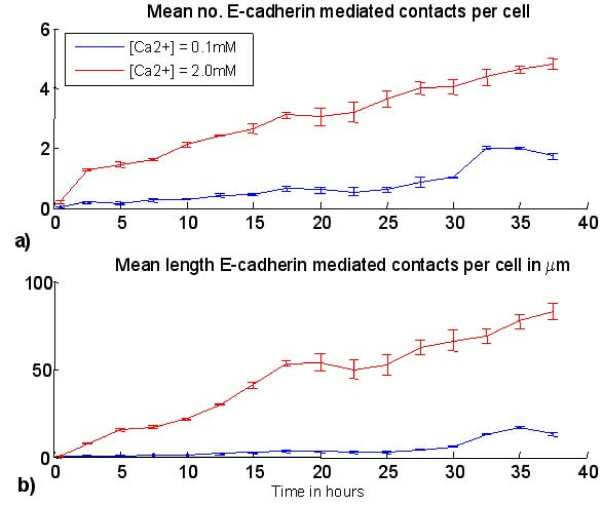
**Agent model results – intercellular contacts**. a) Mean number of E-cadherin mediated contacts per cell predicted by the agent model for a starting cell density of 200 agents/mm and b) mean total perimeter length of each agent associated with E-Cadherin mediated contacts. Vertical lines represent standard deviation calculated from 3 simulations.

**Figure 8:**
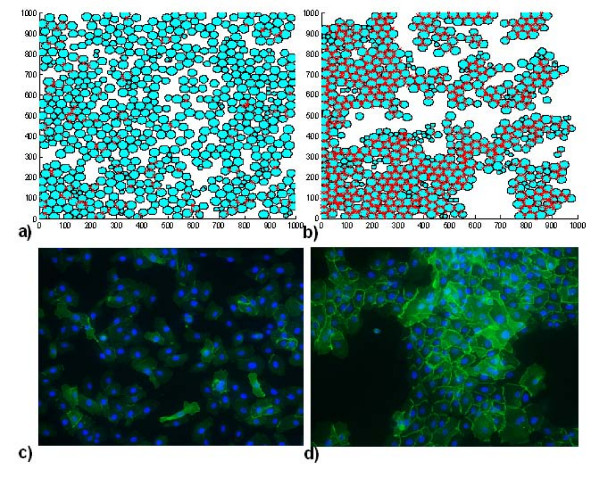
**Distribution of E-cadherin**. a-b) Virtual E-Cadherin immunofluorescence image – 2D snapshot of the agent population (light blue circles) and the E-Cadherin mediated contacts (red lines) after 50 iterations (25 hours) in a) 0.1 mM and b) 2.0 mM extracellular calcium. Original seeding density = 200 cells/mm^2 ^c-d) actual immunofluorescence microscopy images of normal human urothelial cells cultured in 0.09 mM and 2.0 mM [Ca^2+^] conditions, c and d, respectively. Cells were labelled with an antibody that specifically recognises E-cadherin (green), with nuclei counterstained in blue.

The difference in the nature of the contacts formed in the two virtual environments is illustrated in figures 7, 8. Figure 7a shows the relative frequency of E-cadherin mediated contacts in the case of a seeding density of 200 cells/mm^2 ^(2 × 10^4 ^cells/cm^2^). Figure 7b shows the mean length of the perimeter of each cell associated with this type of contact. This demonstrates that even after the simulated cell cultures reach confluence, E-cadherin-mediated contacts are significantly more common and more stable in physiological calcium conditions. Finally, figure 8a–b illustrates a virtual immunofluorescence experiment showing E-cadherin localisation (red lines) in an agent population (blue circles) after simulations have been run in low or high calcium conditions for 50 iterations (25 hours). This can be compared to figure 8c–d, which shows representative immunofluoresence microscopy images of NHU cells (nuclei labelled blue) cultured in low (c) and physiological (d) calcium conditions and labelled with an antibody that recognises E-cadherin protein (green). Virtual time lapse movies of the simulations in low (0.1 mM) and physiological (2 mM) calcium concentrations are available to view in the additional files 3 and 4 respectively.

### Phosphorylated-Erk profile associated with a cell population

**Figure 9:**
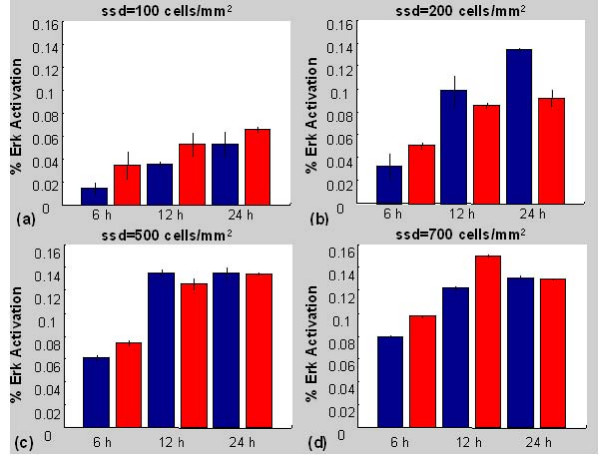
**Virtual Western Blots**. Results of agent simulations in low (blue) and physiological (red) calcium presented in the form of virtual Western Blots. Percentage of total Erk in the activated state over the entire agent population is shown at 6, 12 and 24 hrs after the start of simulations for seeded agent densities (ssd) of (a) 100 cells/mm^2^, (b) 200 cells/mm^2 ^(c) 500 cells/mm^2 ^and (d) 700 cells/mm^2^. The error bars represent 1 standard deviation above and below the mean, obtained from the three independent simulation replicates.

Figure 9 shows the results of a 'virtual' Western Blot assay for populations of interacting agents. Briefly, initial populations of 100, 200, 500 and 700 cell agents were "seeded" on a 1 mm × 1 mm virtual surface and expanded *in silico*, with the model parameter that represents the extracellular calcium concentration set to either 0.1 mM or 2.0 mM. The intracellular pathway model shown in figure 1a was solved 'inside' every agent in direct contact with adjacent neighbours. At every 1 minute time point, the fraction of activated Erk relative to total Erk associated with every agent, was calculated and divided by the total agent number to provide a normalised virtual Erk-PP profile. Each virtual assay was repeated three times to represent inter-experimental replicates and ensure that results were not a product of stochastic effects inherent in the model. As calculations for each agent were carried out individually using a variable time step ODE solver (Matlab ode23tb), each profile was interpolated to provide Erk activation levels at common 1 minute time intervals. Profiles derived at time points representing 6, 12 and 24 hours after the start of the simulation are plotted as bar graphs, equivalent to what is routinely done for *in vitro *experimental immunoblotting data from densitometer plots of the signal.

Although the results from the simple two cell model suggest that Erk activation associated with an individual transient contact is relatively short lived (figure 4), the summation over relatively large number of cells 'cultured' in low calcium medium, continuously making and breaking contacts, leads to continuously elevated Erk-PP. By contrast, in physiological calcium, stable contacts are formed quickly. The consequence of this is that averaged levels of Erk activation measured at fixed time points in cell populations exposed to the two different environments both appear very similar (figure 9), even though the activation levels in individual cells can vary significantly. These results suggest that in populations where the cell density is relatively high, but growth is not contact-inhibited (ie pre-confluent cultures with several hundred cells per mm^2^), the activated Erk signal averaged over the population would be higher in low calcium (e.g. figure 9b), than in physiological calcium environments.

In simulations of very low cell density (figure 9a) intercellular contacts are scarce, and Erk activation is always higher in physiological calcium where any intercellular contacts that occur are maintained for longer durations. For a starting density of 200 cells/mm^2^, the Erk-PP signal in the low calcium simulations exceeds that in the physiological calcium simulations after 12 hours (figure 9b), and this relative elevation persists until at least 24 hours. For seeding densities of 500 and 700 cells/mm^2 ^(figure 9c–d), the population levels quickly approach confluence. In these conditions, space constraints limit cell migration and the opportunity for cells to form transient contacts, and the Erk-PP profiles are similar in both low and physiological calcium environments.

A qualitative, second level sensitivity analysis was conducted for a starting cell density of 200 cells/mm^2^. Five kinetic parameters pertaining to ligand/receptor interactions, five downstream parameters with relatively high sensitivity coefficients and the E-cadherin contact growth rate parameter were systematically varied by a factor of two. The cell culture simulation was run for a period representing 25 hours. Although the amplitudes of the virtual Erk activation level were altered, the prediction of comparable or higher activation levels in the low calcium, relative to the physiological calcium simulations continued to hold true in every case (results not shown).

### Phospho-Erk Immunofluorescence Microscopy Data

Ideally, we would validate our single cell pathway model by tracking Erk activation in cells making different types of contacts under controlled conditions. Unfortunately, this is not possible due to difficulties in visualising protein activation levels and intercellular contact formation in real time. However, our modelling results predict that an otherwise homogeneous population of non-confluent cells sampled from a small spatial region would at any moment in time have a unique pattern of intercellular contacts and hence would be expected to show heterogeneity in Erk activation if examined by immunofluorescence microscopy.

Figure 6a–b shows a snap-shot of the distribution of Erk-PP in urothelial cells maintained in low and physiological calcium visualised by immunofluorescence microscopy. All cells show some degree of Erk activation (indicated by the presence of nuclear Erk), which can be attributed to pathway activation by soluble EGF present in the culture medium. However, in both images there is clear evidence of heterogeneity, with the cells indicated showing low, moderate and high levels of Erk activation, respectively, which suggests that there is a secondary mode of stimulation in these cells.

## Discussion

Our model of juxtacrine signalling between individual cells has yielded two interesting results. At the level of a pair of cells forming a single intercellular contact, the model suggests that the temporal characteristics of activated Erk are highly dependent on the rate of formation and stability of the contact. Specifically, our results suggest that transient contacts result in only transient Erk activation, irrespective of the spatial extent of the contact, whereas more slowly formed, stable contacts stabilised by the interaction of E-Cadherin result in a small but sustained Erk activation, due to the engagement of new receptors and ligands. Secondly, when incorporated into a multi-agent simulation and used to derive an averaged Erk-PP value for the population (analogous to a Western blotting experiment), seemingly contradictory results can be obtained. Specifically, for low density populations (<400 cells/cm^2^), the averaged Erk activation level in low calcium environments (where cells are migratory and most contacts are transient) is equal, or even higher than in physiological calcium environments (where most contacts develop slowly, then remain stable).

Our model predictions relating to Erk activation at the single cell level has potential implications for our understanding of how proliferation through juxtacrine mechanisms is regulated within cell populations. The temporal characteristics of Erk activation – the Erk-PP "signature" – is recognised as a critical factor in determining cell fate. Thus, an initial peak in Erk-PP followed by sustained activation for several hours has been shown to be associated with cell cycle progression, whereas a transient peak followed by a rapid decline is insufficient to induce cycle progression ([12,13]). This suggests that by providing a sustained basal level of Erk activation, juxtacrine signalling via EGFR could provide a mechanism for the increased growth rates that we have previously observed in NHU cells cultured in physiological *versus *low calcium environments [3]. A positive relationship between cell contact and proliferation has also been observed in endothelial and smooth muscle cells [26] and in kidney epithelial cells [27].

The negative association of cell-cell contact and proliferation (contact inhibition of growth) is well accepted. In our agent-based model, this is currently represented by an arbitrary rule that a cell exits the mitotic cycle when it has ≥ *n *bonds with neighbouring cells. At this stage we do not know which other intercellular mechanisms are involved in positive and negative growth modulation, but one candidate is β-catenin, which is a nuclear transcription factor inactivated by E-cadherin sequestration. Future computational and experimental exploration of intracellular processes involving β-catenin that builds on the work of others [28] should indicate whether this is a plausible explanation.

We have not yet fully developed rules for the agent level simulation to relate the proliferation of cells (or more specifically, their decision to pass the G1-S checkpoint) to the Erk-PP profile. This is a natural extension of the model and would allow us to explore how intercellular interactions impact on intracellular molecular state and ultimately on normal and abnormal tissue growth.

By necessity, the model presented here is a simplification of the real intracellular pathway, which may include up to 19,000 distinct molecular species ([29]). We have so far neglected possible membrane diffusion of receptors and ligands, potential targeting of newly synthesised EGFR and ligand to, or clustering at, stable E-cadherin-mediated contacts. We have also ignored the presence of soluble autocrine or exogenous ligands in the medium, which might provide some level of pathway activation independently of any juxtacrine effect (as demonstrated by immunofluorescence in figure 6, where exogenous EGF was present in the growth medium) and other interacting intracellular cascades that may also be downstream of the EGFR (e.g. the P13K-Akt pathway [30]). It is possible that membrane-bound and soluble forms of a ligand may mediate differential effects on EGFR activation. However, sensitivity testing suggests that our basic finding that sustained Erk activation is associated with gradually-formed, stable contacts is not affected by a perturbation in kinetic parameters.

The multi-agent simulations have yielded results that have potential implications for the interpretation of experimental data. The frequency of intercellular contact in a cell population is related to cell density – the greater the number of cells in a given surface area (or volume in 3D culture) the greater the likelihood of contact formation. We have observed that even in low calcium environments, cells can form E-cadherin-mediated adherens junctions in medium to high density cultures/simulations, although these are substantially less common than in physiological calcium (figures 7, 8, [20] and additional files 3 and 4). Simulated cell cultures with initial cell densities of up to the maximum simulated value of 700 cells/mm^2 ^are sub-confluent and remain within the exponential phase of population growth (a 1 mm × 1 mm model reaches visual confluence at approximately 1000 cells). This observation, along with model predictions of the relative frequency and perimeter length associated with E-cadherin mediated cell contact in the different environments (as shown in figure 7), indicates that at high density, cells in low calcium are still able to migrate and make contacts that are predominantly transient in nature (as shown in additional file 2). This suggests that the elevated population-based Erk-PP signal in low calcium simulations (figure 9) represents the summation of multiple transient signals over relatively large cell numbers, rather than an alteration of the nature of intercellular contacts at increased cell density.

These results provide an important insight into the potential pitfalls of interpreting experimental data that is averaged over relatively large cell numbers. Practical limitations mean that by necessity, data is often collected from cell populations and used to draw inferences on individual cell behaviour or intracellular state that cannot be easily observed in the laboratory. A common of example of the latter in a cell biology context is the interpretation of Western blotting data obtained from cultured cell populations. In this case, amounts of a particular protein (active, inactive or total) averaged across a large cell population are measured and used to draw conclusions relating to expression levels in individual cells in the population. We have presented the simulated levels of activated Erk across our entire agent population in the form of a 'virtual' Western blot (figure 9). These model predictions highlight the fact that experimental data obtained from populations of cells should be viewed with caution, particularly in terms of inferring behaviour on the level of the individual cell. The methodology of extracting and analysing our simulated data is directly analogous to the process that an experimental cell biologist would routinely use in the laboratory and highlights the versatility of the agent-based paradigm and its natural relationship to the experimental world.

Our multi-scale model has demonstrated how a) changes in the extrinsic environment affect intercellular contact formation and impact upon intracellular molecular state and b) how the combination of many instances of these states can impact on the measurements that are made on a population level. Clearly, our model is at present simplistic, as it describes a direct causality between EGFR activation, Erk status and proliferation, when in reality, cross-talk from other signalling pathways may modulate at all levels. Nevertheless, the example used to illustrate our novel approach to modelling heterogeneity within populations is biologically-relevant, and this single example of *extrinsic heterogeneity *could be extended to examine other nutrients, growth factors or cytokines in the environment. The second obvious extension of this idea would be to investigate the concept of *intrinsic heterogeneity*, where individual cells in a population differ in terms of their gene expression and numbers or concentrations of particular receptors, ligands or intracellular species. An example of this could be the situation where an E-cadherin^null ^cell arises by mutation and the fate of the population subset is determined relative to its interactions with the rest of the population. This could have important potential implications for understanding how proliferation controls are lost in the event of malignant transformation and how such malignant cells behave in comparison to their normal counterparts during carcinogenesis.

## Conclusion

By combining a deterministic, ODE-based model of a signalling pathway with a non-deterministic agent-based representation of a multi-cellular population, we have developed a simulation environment that can be used to compare how emergent heterogeneity in the cell population can impact on system level behaviour. Our model predicts that the heterogeneity of cell contacts, arising as a result of the inherently stochastic processes of plating cells in culture and the subsequent random migratory behaviour of these cells, provides a basis for heterogeneity in biological response within a 'homogeneous' population. In this paradigm, factors that modulate the random nature of the cell contact may have a major role in influencing the behaviour or phenotype of emergent subpopulations. We have also demonstrated how an agent-based computational approach has a role as an iterative, predictive tool by generating 'virtual assays' to bring new insight into the interpretation of cell biological experiments.

## Authors' contributions

DW developed the models, carried out simulations, analysed the model results and drafted the manuscript. NTG planned and carried out the time lapse microscopy and immunolocalisation experimental studies and analysed the results. JS participated in the design and coordination of the experimental study and helped to draft the manuscript. All authors jointly conceived the study and have read and approved the final manuscript.

## Supplementary Material

Additional file 1 **Time lapse movie of cultured low density urothelial cells in low (0.09 mM) calcium – recording frame rate = 1 frame per minute.** Click here for file

Additional file 2 **Time lapse movie of cultured high density urothelial cells in low (0.09 mM) calcium – recording frame rate = 1 frame per 10 minutes.** Click here for file

Additional file 3 **Virtual time lapse movie of agent simulation in a low calcium (0.1 mM) environment. Circles represent agents (cells) and red lines represent E-cadherin mediated contacts. 1 frame = 30 minutes.** Click here for file

Additional file 4 Virtual time lapse movie of agent simulation in a physiological calcium (2.0 mM) environment. Circles represent agents (cells) and red lines represent E-cadherin mediated contacts. 1 frame = 30 minutes. Click here for file
